# Aging and Exercise Affect Hippocampal Neurogenesis via Different Mechanisms

**DOI:** 10.1371/journal.pone.0132152

**Published:** 2015-07-06

**Authors:** Ting-Ting Yang, Chen-Peng Lo, Pei-Shan Tsai, Shih-Ying Wu, Tzu-Feng Wang, Yun-Wen Chen, Ya-Fen Jiang-Shieh, Yu-Min Kuo

**Affiliations:** 1 School of Chinese Medicine for Post-Baccalaureate, I-Shou University, Kaohsiung, Taiwan; 2 Institute of Basic Medical Sciences, National Cheng Kung University, Tainan, Taiwan; 3 Department of Cell Biology and Anatomy, National Cheng Kung University, Tainan, Taiwan; University of Nebraska Medical Center, UNITED STATES

## Abstract

The rate of neurogenesis is determined by 1) the number of neural stem/progenitor cells (NSCs), 2) proliferation of NSCs, 3) neuron lineage specification, and 4) survival rate of the newborn neurons. Aging lowers the rate of hippocampal neurogenesis, while exercise (Ex) increases this rate. However, it remains unclear which of the determinants are affected by aging and Ex. We characterized the four determinants in different age groups (3, 6, 9, 12, 21 months) of mice that either received one month of Ex training or remained sedentary. Bromodeoxyuridine (BrdU) was injected two hours before sacrificing the mice to label the proliferating cells. The results showed that the number of newborn neurons massively decreased (>95%) by the time the mice reached nine months of age. The number of NSC was mildly reduced during aging, while Ex delayed such decline. The proliferation rates were greatly decreased by the time the mice were 9-month-old and Ex could not improve the rates. The rates of neuron specification were decreased during aging, while Ex increased the rates. The survival rate was not affected by age or Ex. Aging greatly reduced newborn neuron maturation, while Ex potently enhanced it. In conclusion, age-associated decline of hippocampal neurogenesis is mainly caused by reduction of NSC proliferation. Although Ex increases the NSC number and neuron specification rates, it doesn't restore the massive decline of NSC proliferation rate. Hence, the effect of Ex on the rate of hippocampal neurogenesis during aging is limited, but Ex does enhance the maturation of newborn neurons.

## Introduction

Neural stem/precursor cells (NSCs) in the subventricular zone and subgranular zone of dentate gyrus in hippocampus produce new cells throughout adulthood [1,2]. In the hippocampus, the proliferated neuronal progenitor cells migrate into the granule cell layer, then extend dendrites into the molecular layer and project axons to the hilus of the CA3 region [3–5]. These newborn neurons mature and become granular cells, which have demonstrated the ability to modulate the formation of long-term potentiation of the hippocampal circuitries [6]. Recent studies revealed that the formation, exhibition and/or clearance of certain types of hippocampus-dependent memory are influenced by adult hippocampal neurogenesis [7,8].

Hippocampal neurogenesis continues throughout life and is known to be affected by multiple factors. Among these factors, aging is a well-known negative regulator of hippocampal neurogenesis [5,9–12]. In contrast, running exercise (Ex) increases hippocampal neurogenesis during the process of aging [5,13–15]. The rate of adult hippocampal neurogenesis is controlled by the following four determinants: 1) the number of NSCs in the dentate gyrus, 2) proliferation rate of the NSCs, 3) neuronal lineage specification rate of newly proliferated cells, and 4) survival rate of newborn neurons. It is unclear whether aging and Ex act on the same determinant(s) to affect the rate of adult hippocampal neurogenesis or these two factors differentially influence their own targets.

To answer this question, we characterized the four determinants of adult hippocampal neurogenesis in the exercise mouse (Ex) group and the sedentary mouse (Sed) control group, that are further categorized by age into subgroups. We used the stem cell marker, nestin, to label the NSCs and we used bromodeoxyuridine (BrdU), a thymidine analog, to label the proliferating cells [16]. The number of these cells was counted using a modified stereology method. The proliferation rates were calculated by dividing the number of BrdU^+^ cells by the number of nestin^+^ cells. As the newly proliferated cells differentiate into immature neurons, they stop expressing nestin and start to produce doublecortin (DCX) [16]. DCX, expressed in newborn neurons for up to four weeks, is widely used as a marker for immature neurons. The number of BrdU/DCX dual positive (BrdU^+^DCX^+^) cells was considered as the levels of neurogenesis. The neuron lineage specification rate was calculated by dividing the number of BrdU^+^DCX^+^ cells by the number of BrdU^+^ cells. The survival rate of newborn cells was determined by dividing the number of BrdU^+^ cells, at four weeks after BrdU injection, by the number of BrdU^+^ cells at two hours after BrdU injection. We further measured the number of dendrite branches and the dendritic lengths of the DCX^+^ immature neurons as indications of newborn neuron maturation.

## Materials and Methods

### Animals

Male C57BL/6J mice obtained from the Laboratory Animal Center, National Cheng Kung University were used for all experiments. All experimental protocols were performed according to National Institutes of Health guidelines for animal research (Guide for the Care and Use of Laboratory Animals) and were approved by the National Cheng Kung University Institutional Animal Care and Use Committee. The four determinant elements of the adult hippocampal neurogenesis were analyzed at the ages of 3, 6, 9, 12 and 21 months. Six weeks before the due time, half of the animals were subjected to treadmill exercise as described below. Six mice were assigned to each group.

### Treadmill exercise (Ex)

The detailed protocol of Ex training has been described elsewhere [5]. The 5-week Ex training program contained a 1-week familiarization phase followed by a 4-week formal Ex training phase. Mice were first subjected to the familiarization phase to reduce handling and environment-related stimuli. During the familiarization phase, mice were trained to run on the leveled motorized treadmill (Model T408E; Diagnostic & Research Instruments Co., Taiyuan, Taiwan) at a speed of 9 m/min for 10 min/day for five days. Mice were then randomly divided into Ex and Sed control groups. Mice of the Ex group ran at the speed of 10 m/min for 20–60 min/day (an increment of 10 min/day), 5 d/week for the first week, followed by 60 min/day at the same speed, five days per week for the next three weeks. The running speed fulfilled the intensity criterion of moderate exercise [5,15]. Previously, we have shown that this Ex program significantly increased the citrate synthase enzyme activity in soleus muscles, indicating the effectiveness of the Ex training protocol [5,15]. Furthermore, the body weights of mice were significantly decreased after three weeks of Ex training (S1 Fig). Mice of the sedentary (Sed) control groups were placed on a treadmill without running until the Ex group running period has elapsed. A gentle back touch was sufficient to keep the mice running and the entire training process was carried out without electric shock. The front quarter of the treadmill was covered by a dark cloth, creating a dark area that attracts the mice to run forward. The exercise was performed from 1700 to 1800, the last hour in the light cycle, so it would coincide with the mouse circadian activity patterns. Occasionally, mice were excluded from the study because they were not compliant with the training.

### BrdU administration and brain tissue processing

BrdU (Millipore, Temecula, CA; 0.2 mg/g of body weight) was intraperitoneally injected into mice one day after the completion of Ex training program. Two hours or four weeks after the BrdU injection, mice were anesthetized and perfused with ice-cold phosphate buffered saline as previously described [5]. Brains were collected, post-fixed in 4% buffered paraformaldehyde for 48 hours at 4°C, cryoprotected in 30% sucrose solution, sliced into 30 μm sections, and stored in cryoprotectant at -20°C.

### Immunohistochemistry

The procedure for immunohistochemistry has been described previously [5]. The paraformaldehyde fixed brain sections were stained by rabbit anti-nestin (1:250, Millpore, Temecula, CA) for NSCs, mouse anti-BrdU (1:1000, Millipore, Temecula, CA) for newly proliferated cell, goat anti-DCX (1:750, Santa Cruz Biotechnology, CA, USA) for immature neurons. Brain sections were incubated with appropriate biotinylated secondary antibodies and avidin-biotin peroxidase (ABC, Vector, Burlingame, CA). The sections were visualized by reaction with 3,3′-diaminobenzidine using nickel-enhanced method [5]. For BrdU staining, brain sections were pretreated with 2 N HCl at 37°C for 30 minutes to denature the DNA, followed by neutralization with 100 mM sodium borate incubation for 5 minutes.

For BrdU^+^DCX^+^ double immunohistochemical labeling, free-floating tissues were first immunostained with mouse anti-BrdU (1:1000, Millipore) and developed by reaction with 3,3′-diaminobenzidine using nickel-enhanced method (deep purple). The procedure was followed by second labeling with goat anti-DCX (1:750, Santa Cruz Biotechnology), then visualized by reaction with 3,3′-diaminobenzidine only (brown).

### Cell counting

A modified stereology protocol was used to quantify labeled cells [5]. The entire hippocampal dentate gyrus area was cut into an average of 96 coronal sections with a thickness of 30 μm. The number of Nestin^+^ cells, that of BrdU^+^ cells and that of DCX^+^ cells were counted in every sixth section. To prevent observer bias, sections were coded to ensure that the observer was blind to their condition. Positive cells were counted through the 40X objective under a Ziess microscope. The total number of labeled cells per section was determined and divided by the slide selection ratio (i.e. 16/96), to obtain the total number of labeled cells in each dentate gyrus [5].

### Quantification of dendritic branch and length of DCX+ newborn neurons

The dendritic branch number and length of DCX^+^ cells were selected to estimate the maturation rate of newborn neurons. Only those DCX^+^ cells showing minimal dendritic tree overlap with adjacent cells were included to avoid ambiguity [5]. By using an Axiocam MRc digital camera connected to a computer equipped with Axiovision 4.8 software (Carl Zeiss, Inc., Thornwood, NY), photomicrographs were taken through the 40X objective under a Ziess microscope. The dendritic tree was traced manually and the dendritic branch points were counted. The dendritic lengths were analyzed using the traced dendritic tree by National Institutes of Health Image software (http://rsb.info.nih.gov/ij). The observer was blind to the experimental condition. Ten neurons in 10 brain slices obtained from each animal were measured. Five animals in each group were analyzed (n = 5 for each group).

### Statistical analysis

Data are presented as mean ± standard error. Two-way ANOVA was used to analyze the two main effects (age, Ex) and their possible interaction. Bonferroni post-hoc tests were performed if significant (*p* < 0.05) interactions were found.

## Results

### Effects of aging and Ex on the rate of adult hippocampal neurogenesis

**Fig 1:**
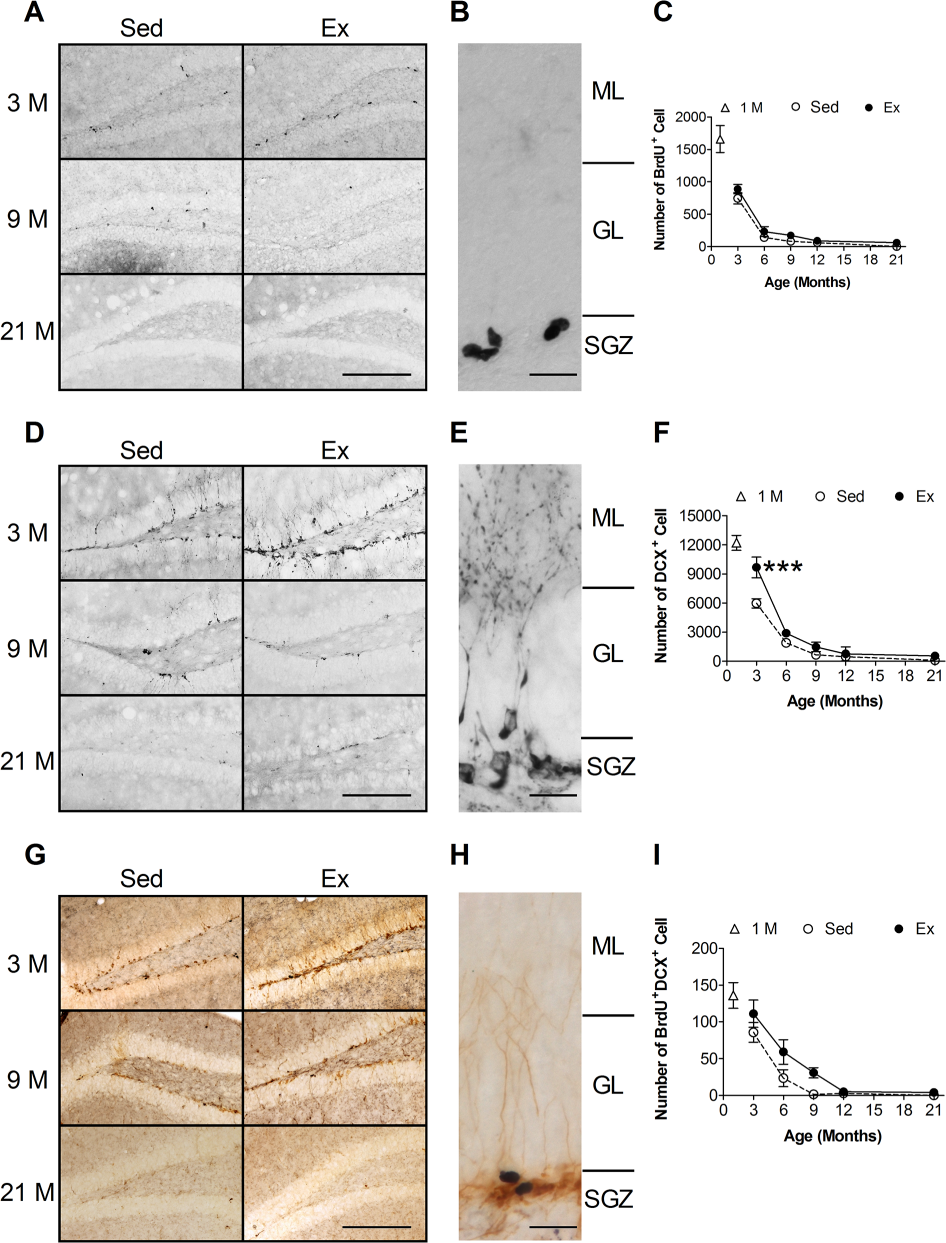
Effects of age and running exercise (Ex) on the number of newly proliferated cells and that of immature neurons in the adult hippocampus. Representative micrographs of BrdU^+^ newborn cells (**A**), DCX^+^ immature neurons (**D**) and BrdU^+^DCX^+^ newborn neurons (**G**) in the hippocampal dentate gyrus of mice at 3, 9 and 21 months of age. Sed: sedentary group. Bar = 100 μm. Enlarged micrographs of BrdU^+^ cells (**B**), DCX^+^ immature neurons (**E**) and BrdU^+^DCX^+^ newborn neurons (**H**) are presented to illustrate the detail locations of these cells. Bar = 20 μm. SGZ: subgranular zone; GCL: granular cell layer; MoL: molecular layer. Quantitative analyses of BrdU^+^ cells (**C**), DCX^+^ cells (F) and BrdU^+^DCX^+^ cells (I) in dentate gyrus of mice with different ages. ***: Bonferroni post-hoc test: p < 0.001 vs. respective Sed group.

To estimate the rate of hippocampal neurogenesis during aging, we counted the BrdU^+^DCX^+^ cell number in the hippocampal dentate gyrus of mice at the ages of 3, 6, 9, 12 and 21 months. Before obtaining the number of BrdU^+^DCX^+^ cells, we collected the number of BrdU^+^ cells and that of DCX^+^ cells first. We injected mice with BrdU to label the newly proliferated cells one day after completion of the 5-week Ex training program. Because the half-life of BrdU is about two hours [17], mice were killed 2 h after injection. As shown in Fig 1A, BrdU^+^ cells mainly resided in the subgranular zone of the dentate gyrus. The BrdU^+^ cells were frequently clustered together, indicating that they might be derived from the same ancestor NSC (Fig 1B). Two-way ANOVA results showed that both age (F = 97.0, d.f. = 4/80, *p* < 0.001) and Ex (F = 6.4, d.f. = 1/80, *p* = 0.013) altered the number of BrdU^+^ cells, without interaction between these two factors (F = 0.4, d.f. = 4/80, *p* > 0.5)(Fig 1C).

The cell bodies of DCX^+^ newborn neurons were mainly located adjacent to subgranular zone (Fig 1D), while the dendrites of DCX^+^ newborn neurons projected into the granular cell layer or crossed the granular cell layer into the molecular layer (Fig 1E). Both age (F = 294.3, d.f. = 4/70, *p* < 0.001) and Ex (F = 44.9, d.f. = 1/70, *p* < 0.001) affected the number of DCX^+^ newborn neurons. However, a significant interaction between these two factors (F = 15.0, d.f. = 4/70, *p* < 0.001) was evident. Bonferroni post-hoc tests indicated that there were differences between the 3-month-old Ex and the 3-month-old Sed groups (Fig 1F).

As shown in Fig 1G, the number of BrdU^+^DCX^+^ cells decreased as age increased. Only cells that showed BrdU^+^ stains in the nucleus and DCX^+^ stains in the cytoplasm (Fig 1H) were counted. Two-way ANOVA revealed that age (F = 33.4, d.f. = 4/70, *p* < 0.001) and Ex (F = 7.2, d.f. = 1/70, *p* = 0.009) significantly influenced the number of BrdU^+^DCX^+^ cells. No interaction between these two factors was evident (F = 0.9, d.f. = 4/70, p = 0.474) (Fig 1I). Compared to 3-month-old mice, the number-s of BrdU^+^DCX^+^ cells decreased to about 27% in the 6-month-old mice. By the time the mice reached 9 months of age, the number of BrdU^+^DCX^+^ cells was only 1.5% of the cell number in the 3-month-old mice (Fig 1I). These results showed that the decline of adult hippocampal neurogenesis mainly occurred before middle age.

To further investigate the age effect on neurogenesis, we have added a group of 1-month-old mice (Fig 1C, 1F and 1I, open triangles). These mice were in pre-puberty stage and could not perform the 5-week Ex training; hence they were not included in the analysis of adult neurogenesis. However, the results of the 1-month-old group suggested that the decline of hippocampal neurogenesis begins at a much younger age (Fig 1C, 1F and 1I).

### Effects of aging and Ex on the number of NSC in the adult hippocampus

**Fig 2:**
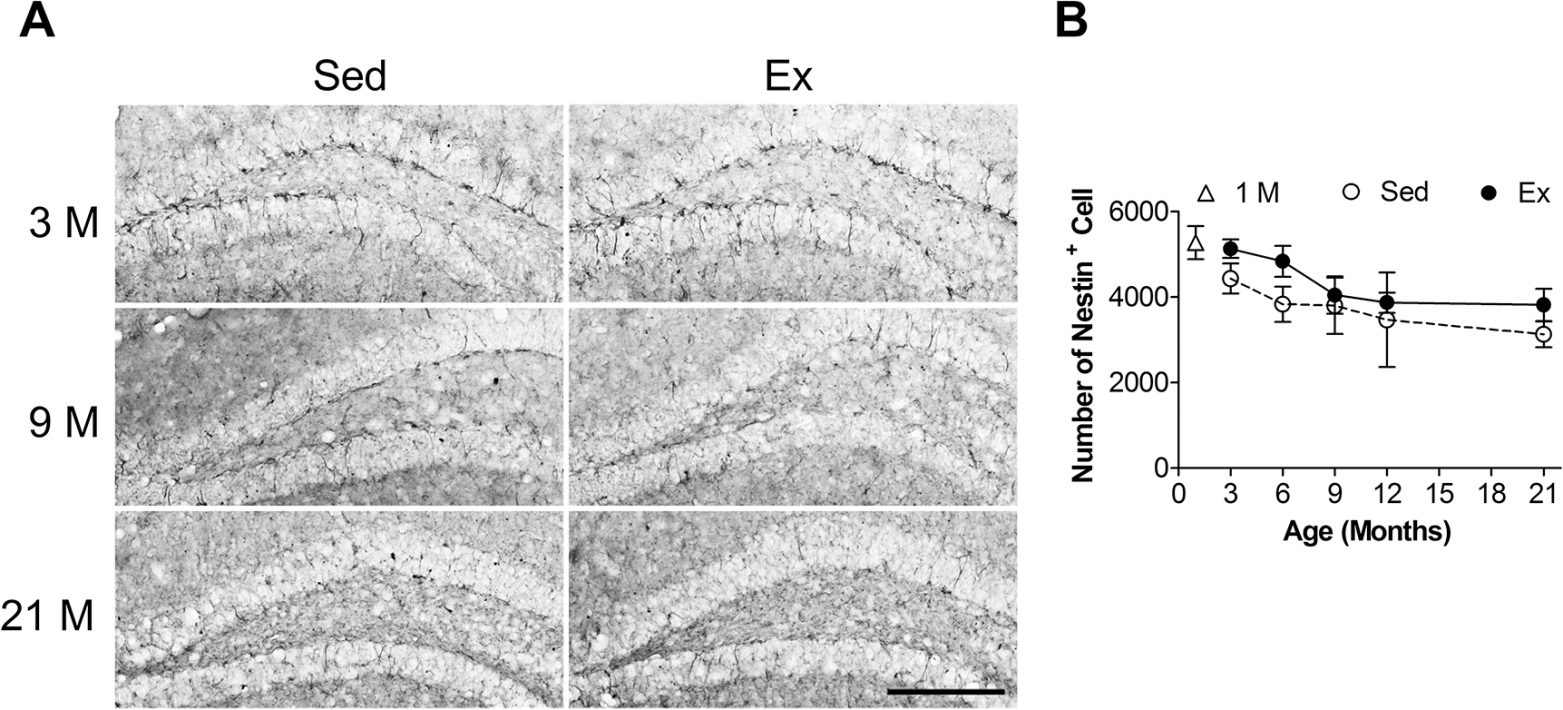
Effects of age and running exercise (Ex) on the number of neural stem/precursor cells (NSCs) in the adult hippocampus. **A**) Representative micrographs of nestin^+^ cells in the hippocampal dentate gyrus of mice at 3, 9 and 21 months of age. Sed: sedentary group. Scale bar: 100 μm. **B**) Quantitative analyses of nestin^+^ cells in the dentate gyrus of mice with different ages.

The effects of aging and those of Ex on NSCs were determined by counting the nestin^+^ cell number in the hippocampal dentate gyrus of mice at the ages of 3, 6, 9, 12 and 21 months (Fig 2A). Two-way ANOVA revealed that both age (F = 4.5, d.f. = 4/44, *p* = 0.004) and Ex (F = 4.5, d.f. = 1/44, *p* = 0.040) altered the number of NSC, without significant interaction between these two factors (F = 0.2, d.f. = 4/44, *p* > 0.5)(Fig 2B). However, post-hoc analysis indicated that the age effect was mainly contributed by the 3-month-old group, while the number of NSC remained unchanged in other older age groups (F = 1.4, d.f. = 3/29, *p* = 0.276). Using 3-month-old mice group as a reference point, the decline of NSCs by the age of 9-month was insignificant, where the measured value was slightly more than 10% (Fig 2B).

We have also estimated the number of NSC of the 1-month-old mice (Fig 2B, open triangle). A weak trend of decline of the number of NSC was evident beginning at an early age.

### Effects of aging and Ex on proliferation, neuron lineage specification and survival rate of adult hippocampal neurogenesis

**Fig 3:**
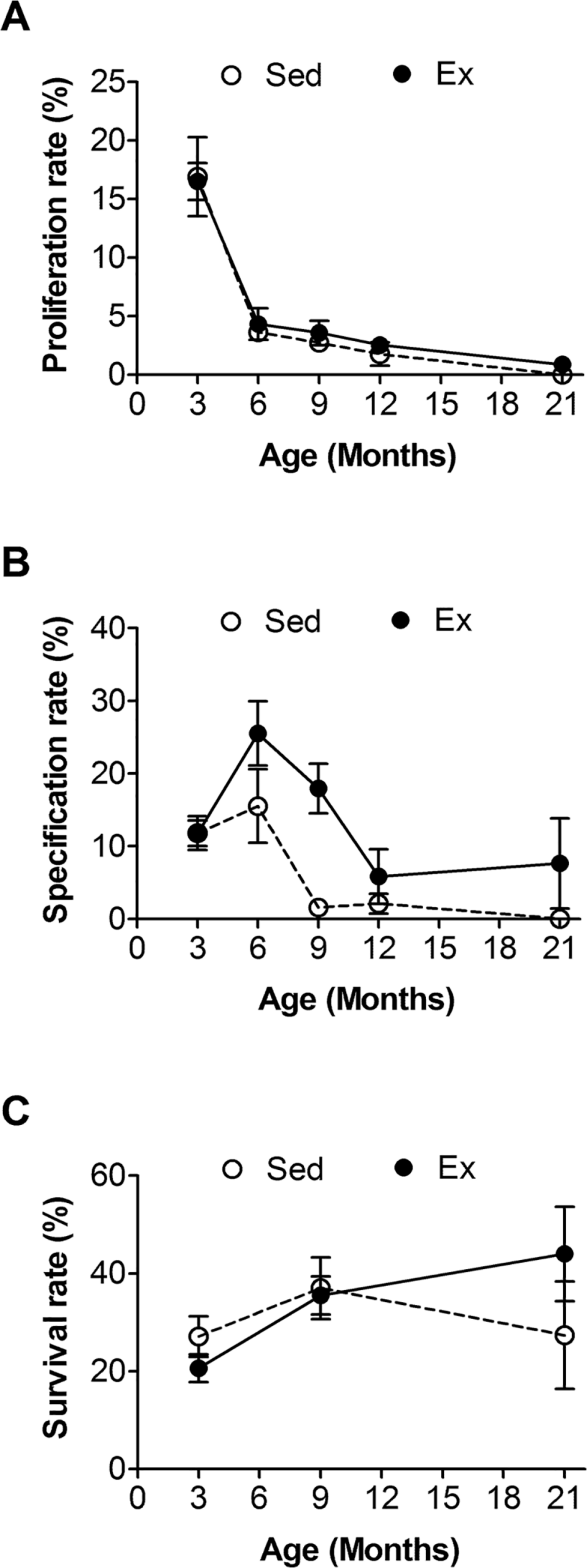
Effects of age and running exercise (Ex) on the proliferation rate, neuron lineage specification rate and survival rate of adult hippocampal neurogenesis. **A**) Proliferation rate, estimated by dividing the number of BrdU^+^ cells by the number of nestin^+^ cells. **B**) Neuron lineage specification rate, estimated by dividing the number of BrdU^+^DCX^+^ cells by the number of BrdU^+^ cells. **C**) Survival rate, estimated by dividing the number of BrdU^+^ cells, 2 h after BrdU injection, by the number of BrdU^+^ cells 1 month after BrdU injection.

The number of newly proliferated cell is determined by the number of stem cells and the rate of stem cell proliferation. Therefore, we calculated the proliferation rate by dividing the number of BrdU^+^ cells by the number of nestin^+^ cells. The results showed that age (F = 42.0, d.f. = 4/61, *p* < 0.001), but not Ex (F = 0.3, d.f. = 1/61, *p* > 0.5), significantly changed the proliferation rate (Fig 3A). There was no interaction between the two factors (F = 0.1, d.f. = 4/61, *p* > 0.5). The proliferation rates of the 6-month-old and the 9-month-old mice were about 22% and 16% of the rate of the 3-month-old mice, respectively (Fig 3A).

Among the newly proliferated cells, only a certain proportion of them become neurons (lineage specification). Therefore, we determined the neuron lineage specification rate by dividing the number of BrdU^+^DCX^+^ cells by the number of BrdU^+^ cells. However, due to the short (2 h) post-BrdU injection interval, we failed to detect BrdU^+^ cells in some aged mice. These mice were excluded from the specification rate analysis. The results revealed that age (F = 5.4, d.f. = 4/55, *p* = 0.001) and Ex (F = 10.3, d.f. = 1/55, *p* = 0.002) had significant effects on the neuron lineage specification rate (Fig 3B). No interaction was found between these two factors (F = 1.8, d.f. = 4/55, *p* = 0.137). The lineage specification rate of the 9-month-old mice was about 14% of that of the 3-month-old mice (Fig 3B).

Most of adult newborn neurons do not survive a few weeks after birth [5,18]. Therefore, the survival rate contributes greatly to adult neurogenesis. The survival rate was determined by dividing the number of BrdU^+^ cells in the mice, four weeks after receiving BrdU injection, by the number of BrdU^+^ cells in the mice two hours after BrdU injection. We only estimated the survival rates of mice in the 3, 9 and 21-month-old groups, which represent young, middle-age and old age mice, respectively. Two way ANOVA revealed that neither age (F = 1.3, d.f. = 2/47, p = 0.279) nor Ex (F = 0.2, d.f. = 1/47, p > 0.5) had any effect on survival rate. There was no interaction between the age and Ex factors (F = 1.2, d.f. = 2/47, *p* = 0.320) (Fig 3C).

### Effects of aging and Ex on the maturation of newborn neurons

**Fig 4:**
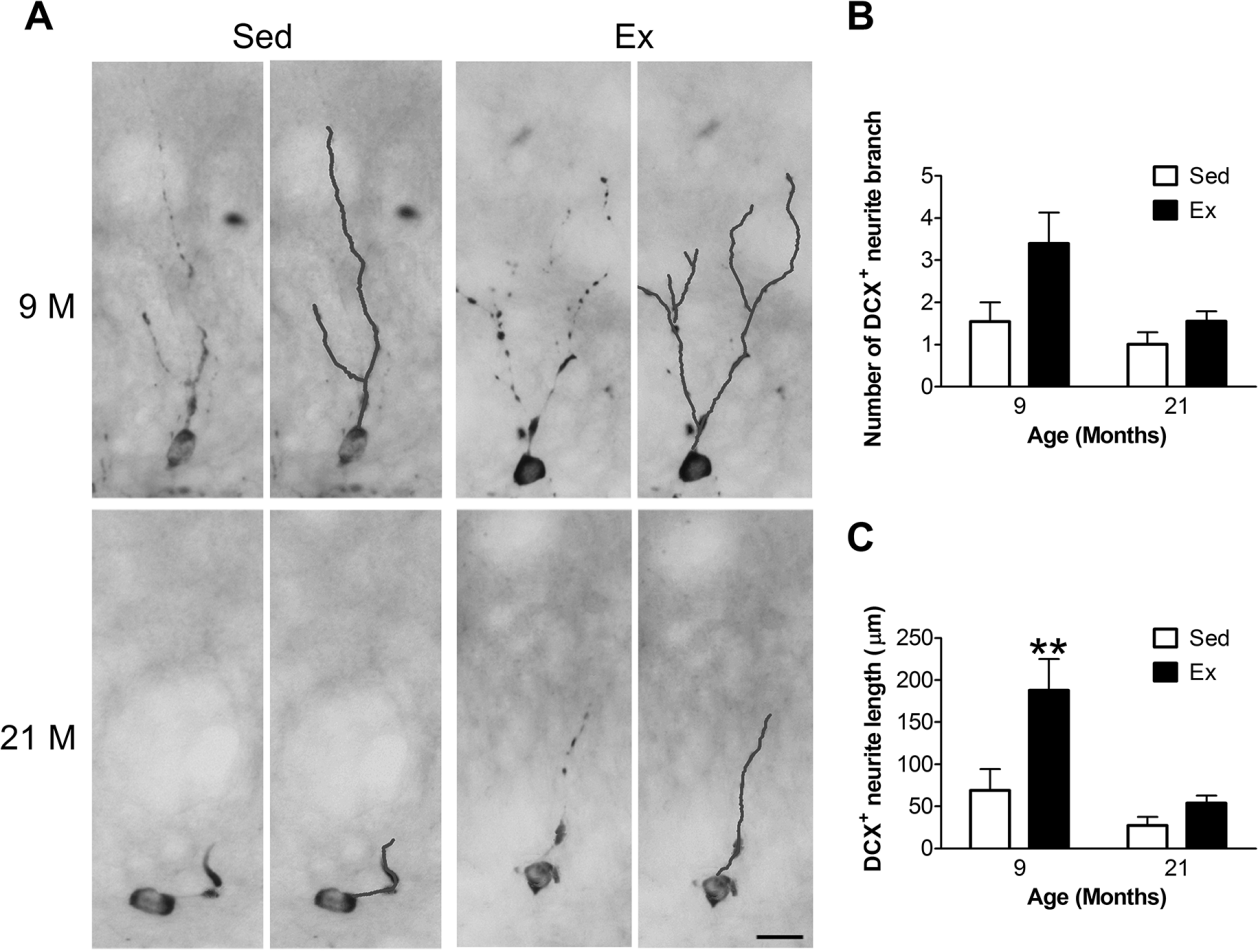
Effects of age and running exercise (Ex) on the maturation of newborn neurons. **A**) Representative micrographs of DCX^+^ immature neurons in the dentate gyrus of the 9 and 21-month-old mice that received or did not receive Ex training. Examples of tracings (blue line) are used to measure the quality of dendrite of DCX^+^ immature neurons. Sed: sedentary group. Scale bar: 20 μm. **B**) Quantitative results of the number of branches of DCX^+^ dendrite. **C**) Quantitative results of the total length of DCX^+^ dendrite. **: Bonferroni post-hoc test: p < 0.01 vs. respective Sed group.

We used the dendritic branch number and length of the DCX^+^ cells as an indication of newborn neuron maturation (Fig 4A). Because the dendritic arbors of the DCX^+^ cells substantially overlapped with neighboring DCX^+^ cells in young age mice, and thus interfered with analysis, we only compared the dendritic complexity of DCX^+^ cells in mice of 9- and 21-month-old groups. The number of dendritic branches was significantly affected by age (F = 6.4, d.f. = 1/16, *p* = 0.022) and Ex (F = 7.2, d.f. = 1/16, *p* = 0.016), with no interaction between these two factors (F = 1.8, d.f. = 1/16, *p* = 0.195) (Fig 4B). Similarly, the dendritic lengths were affected by age (F = 17.6, d.f. = 1/16, *p* < 0.001) and Ex (F = 12.5, d.f. = 1/16, *p* = 0.003). However, a significant interaction was observed between the two factors (F = 4.8, d.f. = 1/16, *p* = 0.044). Bonferroni post-hoc tests indicated that the dendritic lengths of the 9-month-old Ex mice were significantly longer than those of the 9-month-old Sed mice (Fig 4C).

## Discussion

We hypothesized that the levels of neurogenesis in adulthood were determined by the number of NSC, proliferation of NSC, neuron lineage specification of newborn cells, and survival rate of newborn neuron. We used these four elements to evaluate the effects of aging on adult hippocampal neurogenesis. Our results showed that the numbers of adult hippocampal neurogenesis decreased remarkably before middle age. By the age of 9 months, the number of newborn neurons in the hippocampus was less than 2% of that of the 3-month-old group. The big drop of neurogenesis during aging could not be explained by loss of NSC, as the number of NSC only marginally declined format the age of 3 months to the age of 9 months. However, the rates of NSC proliferation were largely reduced by about 85% during this time frame. Although the neuron lineage specification rate was also heavily decreased during this time frame, this step occurs after the cell proliferation stage. Therefore, we conclude that age-associated decline of adult hippocampal neurogenesis is caused by decline of NSC proliferation.

One month of running exercise increased the levels of adult hippocampal neurogenesis. The degree of Ex effect on adult hippocampal neurogenesis was similar across different age groups (i.e. no interaction between Ex and age). Ex slightly increased the number of NSC and the rates of neuron lineage specification, without changing the rates of NSC proliferation. Furthermore, Ex increased maturation of newborn neurons without affecting their survival rate. Thus, Ex-increased adult hippocampal neurogenesis is likely derived from sustaining the number of NSC and increasing the rates of neuron lineage specification. Interestingly, Ex showed a strong effect on promoting the maturation of newborn neurons. These results were in agreement with previous findings that suggests running exercise potently increases the neuroplasticity (e.g. levels of synaptic proteins, complexity of dendritic arbor, density of dendritic spine, etc.) of post-mitotic neurons in several brain regions [19–21].

There are numerous reports showing that running exercise increases NSC division and number of newborn neuronal progenitor cells in rodents [5,13,14,22]. These studies used exogenous BrdU or endogenous Ki67 to label the newly proliferated cells, and used the number of these cells as indications of division. Both numbers were increased by running exercise [5,13,14,22]. Our results (Fig 1A) also supported such findings. However, the number of BrdU^+^ cells was influenced by the number of NSC and the proliferation of NSC. When we adjusted the proliferation rate by dividing the number of BrdU^+^ cells by the number of nestin^+^ NSCs, the effect of Ex became insignificant. That is, the Ex-induced increase in the number of BrdU^+^ cells was mainly derived from the increased number of NSC. Therefore, we suggest that maintaining the NSC number is, at least in part, contributing to the positive effect of Ex on hippocampal neurogenesis during the process of aging.

Whether the number of hippocampal NSC decreases with age is a debated issue. The number of nestin^+^ NSC in the hippocampi of Fischer F344 rats was found to have greatly reduced by the time the rats were in middle age (10-month-old vs. 3-month-old) [23]. However, in another study using Sox-2 to mark NSCs in the hippocampi of young (4-month-old), middle-aged (12-month-old) and aged (24-month-old) F344 rats, the number of NSC did not diminished with age [24]. Using FABP7, Sox2 and GFAP triple stain to label NSCs, the number of hippocampal NSC decreased in the aged monkeys (22-year-old vs. 5-year-old), but not in aged ICR mice (2-year-old vs. 6-week-old) [25]. The authors hypothesized that age-related neurobiological changes were more advanced in the long life-span of primates, and might not be observed in the much shorter life-span of rodents. Furthermore, the NSC number and proliferation rate appear to be species-specific. The numbers of Ki-67^+^ proliferating cells varied greatly among different species of rodents [26]. Furthermore, NSCs isolated from mice and rats responded differently after the treatment of mitogenic growth factors or differentiation factors [27].

Regardless of the species-specific phenomenon, all evidence agrees that the number of newborn neurons in the hippocampus declines with age, especially before middle age. Our findings further revealed that even if the number of hippocampal NSCs has dropped, the trend was modest and only contributed slightly to the reduction of hippocampal neurogenesis during the process of aging. Rather, the remarkable decline of hippocampal neurogenesis during the process of aging is dictated by reduced proliferation of NSCs, a phenomenon that is associated with increased quiescence and decreased self-renewal ability of NSCs [24]. Our results also showed that 1 month of Ex did not revive the quiescent NSCs in aged animals. Thus, Ex-enhanced adult hippocampal neurogenesis is also restricted by low proliferation rates of the NSCs during the process of aging.

Neuron lineage specification rate decreased with age, but could be increased by Ex. In this study, the neuron lineage specification rate was calculated by dividing the number of BrdU^+^DCX^+^ cells by the number of BrdU^+^ cells. Because the animals were killed 2 h after BrdU injection, while the expression of DCX usually begins several days after the birth of a cell, such a short interval may only allow a fraction of the total BrdU^+^DCX^+^ cells to be detected. In other words, the neuron lineage specification rate might be underestimated in this setting. Although longer intervals would allow more accurate estimations of the number of BrdU^+^DCX^+^ cells, the number of newborn neurons might also be confounded by the survival rate.

## Conclusions

Adult hippocampal neurogenesis decreased steeply at a young age in mice. When mice reached 9 months of age, their neurogenesis capability was less than 5% of that of 3-month-old mice. The age-associated decline of hippocampal neurogenesis was not caused by diminished NSC number, but primarily by the reduced proliferation of NSCs. Ex increased adult hippocampal neurogenesis via maintaining the number of NSC and increasing the rates of neuron lineage specification. However, as Ex did not prevent the sharp decline of the NSC proliferation rate, the ability of Ex on restoring adult hippocampal neurogenesis during aging is limited. However, Ex did show a more pronounced effect on enhancing newborn neuron maturation.

## Supporting Information

S1 Fig Effect of running exercise (Ex) on the body weights of different ages of mice. Sed: sedentary group. Bonferroni post-hoc test: * (p < 0.05), ** (p < 0.01), *** (p < 0.001) vs. respective Sed group. The standard deviations are too small to be seen in the scale setting.
(TIF)
